# Healing synchrony? potential benefits of interpersonal synchrony for chronic pain management

**DOI:** 10.3389/fpain.2025.1463321

**Published:** 2025-02-05

**Authors:** Justyna Świdrak

**Affiliations:** ^1^ Systems Neuroscience, Institut d’Investigacions Biomèdiques August Pi I Sunyer (IDIBAPS), Barcelona, Spain; ^2^ Institute of Psychology Polish Academy of Sciences (IP PAS), Warsaw, Poland

**Keywords:** fibromyalgia, bodily self, chronic pain management, interpersonal neural synchrony, interbrain coherence

## Abstract

Fibromyalgia is called a pathology of misconnection at the neurophysiological, psychological, and social levels, and is characterised by widespread musculoskeletal pain, which is accompanied by a series of symptoms, such as chronic fatigue, depression, anxiety, body perception disturbances, and cognitive deficits. In this article, I argue that interventions that in various ways enhance interpersonal neural synchronisation (INS) may bring long-term benefits to people with fibromyalgia (PwF). In the first part, I briefly introduce studies on INS in the general population. In the second part, I hypothesise that interpersonal synchrony may contribute to symptom reduction for individuals with fibromyalgia, in the sense that repeated experience of being in sync with others may play a role in restoring both the brain-body and self-others connection in this population and consequently result in simultaneous lasting improvement of wellbeing. In the final part, I discuss potential future research directions.

## 1 Introduction

With one in ten adults diagnosed each year (1), chronic pain is one of the major health challenges in the world. It has a detrimental effect not only on the wellbeing of those who suffer from it (2), but also on their social and family environment (3, 4). These consequences are evident in fibromyalgia, the most common central sensitivity syndrome, characterised by a combination of symptoms known as FIBRO: Fatigue and Fog (cognitive dysfunction), Blues (depression, anxiety) Rigidity (stiffness), and “Ow!” (chronic pain, tenderness) (5). It is “a pathology of misconnection” at the neurophysiological, psychological, and social levels. Perrot (6) observed that due to its invisibility and lack of a specific biomarker, people with fibromyalgia (PwF) are disconnected from society. They are also disconnected internally due to the complex pathophysiology that leads to a desynchronisation of the brain and body. Consequently, the recommended treatments aim to restore good connections (6). Essential pain symptoms are related to augmented sensory processing (central sensitisation) and an inability to modulate pain effectively (7). I argue that interventions that in various ways enhance interpersonal neural synchronisation (INS) may facilitate a simultaneous body-brain and self-other reconnection and bring long-term benefits to PwF. In this paper, I will briefly introduce studies on INS in the general population and explain its potential role in the treatment of widespread chronic musculoskeletal pain in the context of existing evidence.

## 2 Synchronised brains in synchronised bodies

**Figure 1:**
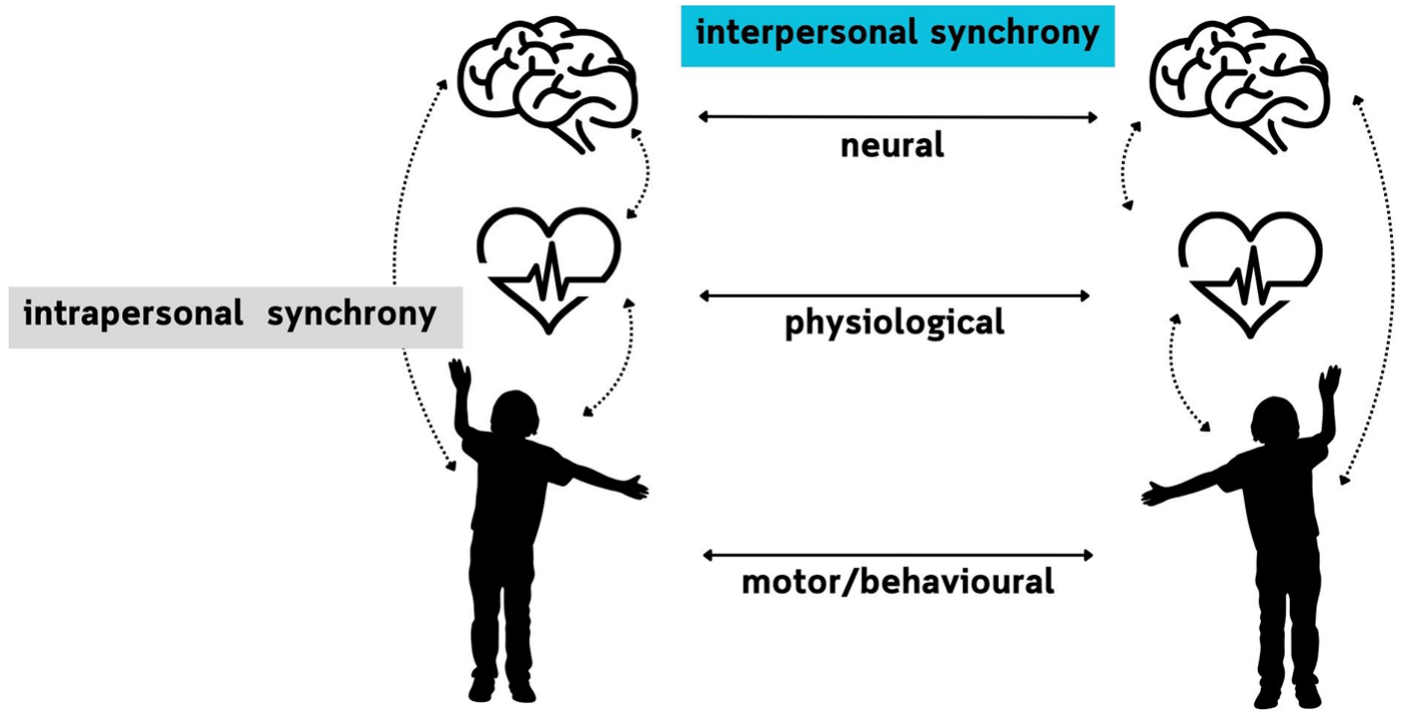
The interplay between the intra- and interpersonal synchrony.

After studying human cognition in isolated individuals (single-brain studies), in the last two decades the interest of social neuroscientists has been shifting towards second-person neuroscience thanks to advances in hyperscanning. This is a simultaneous measurement of more than one person's neural activity (dual-brain studies) (8). Hyperscanning studies led to a discovery of INS which can be defined as a phenomenon “that promotes social interactions by enabling functional integration of multiple brains” (9, 354). Despite its short history, second-person neuroscience has already provided sophisticated theoretical frameworks which attempt to explain the interplay of inter- and intrabrain networks, for example, the hypothesis of hyperbrain cell assembly (10), the plasticity of the interbrain (11), the two-body joint forward model for interpersonal action coordination (12). Another interesting approach is the embodied mutual prediction theory which states that the coherence of neural signals between two brains can arise during a social interaction between two people as their brains simultaneously control own actions and mutually predict each other's actions (13). Importantly, interbrain coherence should not be investigated in isolation from the body, but as a process simultaneously happening on behavioural, psychophysiological, and neural level. These levels need to be analysed jointly, as visual, auditory, and motor processes mediate any coordination between two brains (Figure 1) (14).

**Figure 2:**
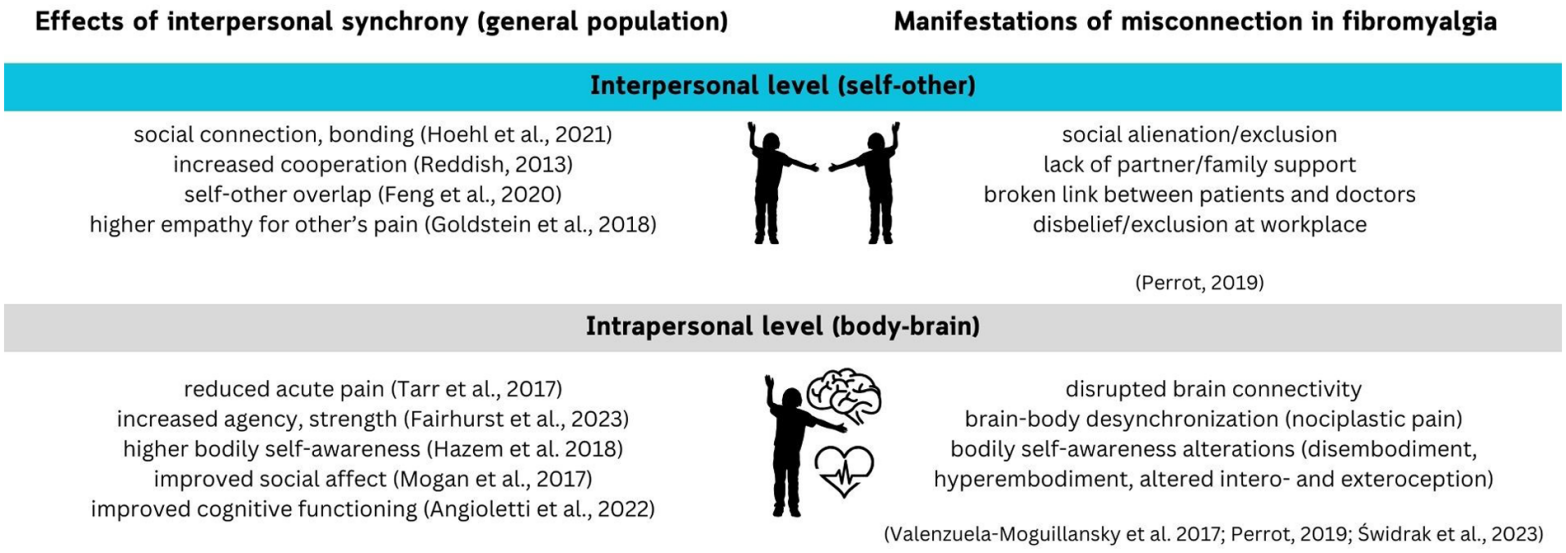
Effects of interpersonal synchrony on typical adults (left) compared to misconnection observed in fibromyalgia (right).

Syncing with others leads to an increased predictability of incoming signals which offers many benefits ranging from basic information processing at the individual level to the bonding of dyads and larger groups (15, 16). Furthermore, synchrony increases cooperation (17), social connection, and improves affect (18). It is associated with self-other overlap (19)*,* which also reduces sensitivity to own bodily pain (20) and increases empathy for the pain of others (21). Interpersonal synchrony during touch can potentially reduce acute pain (22). Thus, synchrony tends to feel good and creates a sense of connection (23). I argue that looking at complex chronic pain conditions such as fibromyalgia through a lens of embodied INS offers an exciting opportunity to reinterpret existing evidence and open new research pathways. I hypothesise that interpersonal synchrony can be healing for PwF, in the sense that repeated experience of being in sync with others may play a role in restoring both the brain-body and self-others connection in this population and consequently result in simultaneous lasting improvement of several key symptoms, such as pain, depression, anxiety, and attention deficits (Figure 2).

## 3 Social bodily self

Similarly to the second-person social neuroscience, a recent shift from the first- to the second-person perspective of the bodily self can be observed (24, 25). The bodily self originates from the multisensory integration of bodily-related signals, with the motor systems playing a key role (26). The social self links the bodily self with that of others (27). It plays a fundamental role in the constitution of the other as another self. It seems that this connection is bidirectional, as there is evidence that social contact enhances bodily self-awareness (28, 29). At the same time, interpersonal synchrony is also known to blur self-other boundaries (30), which may lead to the weakening of affective self-regulation (23). This perspective helps to understand the relationships between a broken brain-body connection, as observed in PwF (31) and the social alienation of this population (3).

According to the sensorimotor theory of pain, changes in sensorimotor processing, especially motor deficits, sensory changes, and body representation distortions, may be a reason for persistent pain (32). Chronic pain often includes not only continuous nociceptive stimulation, but also multiple sensorimotor distortions that lead to a disrupted body experience (33), including distortions in body image (perceiving own body as a physical object with its size and shape) and body schema (representation of a body for action planning and control) (34). Pain is a fundamentally social experience, because it challenges basic human needs, such as the need for autonomy, the need to belong, and the need for justice/fairness (35). People diagnosed with fibromyalgia indeed often struggle with fulfilling these needs as they are commonly alienated, discriminated, and disbelieved by others when discussing their symptoms. This experience of a broken social bodily self manifests in perceiving their bodies as a barrier to the social world, leading to a disruption of a basic sense of being with others (36).

Simple social contact, such as direct gaze, improves bodily self-awareness (28, 29). Furthermore, focussing on its one's own physiological processes during a socially framed task that requires motor synchronisation “boosts” the hemodynamic correlates in the regions of the brain that support sustained attention, reorientation of attention, social responsiveness, and synchronisation (37), cognitive functions often disturbed in fibromyalgia (38, 39). One of the best examples of a recurring embodied social interaction aimed at improving the broken body-mind and social connections is psychotherapy. Therapeutic alliance – the bond between the therapist and the patient/client built throughout the therapeutic process – is one of the main drivers of therapy success (40). There is a growing body of evidence that this success positively correlates with movement synchrony between therapists and patients (41). Furthermore, a review by Sened, Zilcha-Mano, and Shamay-Tsoory (42) indicates that high interbrain coherence is associated with better relationships in both therapy and in daily life, while deficits in the ability to achieve interbrain synchrony are associated with a variety of psychological and developmental disorders. Affective symptoms, such as depression and anxiety, are extremely common in fibromyalgia, and psychotherapy is a central element of many interdisciplinary treatment programs (43). Indeed, evidence indicates that various psychotherapeutic treatments obtain positive effects in short-, mid-, and even long-term (44–48). Interestingly, a comparison of group therapy alone and multidisciplinary programs which contained group therapy demonstrated that the latter are only slightly more effective in reducing primary and secondary symptoms of fibromyalgia (49).

Following this pathway, we can speculate that social interactions that simultaneously promote spontaneous synchronisation with others and a concentration on own bodily signals, such as psychotherapy, yoga and other mind-body activities, may facilitate reconnecting brain-body processing, particularly in the motor and prefrontal areas (50).

## 4 Benefits of moving together

The broken connection between PwF and their social networks often leads to loneliness and depression, and therefore more social and family-orientated research initiatives are recommended (4). Regular physical activity is one of the pillars of the treatment of fibromyalgia, due to its multiple positive influences, including improved mood and sleep quality, raising pain thresholds, breaking the vicious cycle of pain-inactivity-pain, and reducing physical deconditioning (51, 52). Thus, the only “strong” EULAR^1^ recommendation for PwF is exercise (53). Adherence to exercise programmes in chronic pain treatment is the key to their efficiency, but it varies significantly (54), and can be improved by implementing a social component in the programme (55).

One of the most beneficial types of physical activity for PwF is dance. Dance reduces musculoskeletal pain and dance therapy is recommended as an effective addition to the treatment of chronic pain (56), including fibromyalgia, with PwF who dance report lower depression, anxiety and kinesiophobia (fear of movement), and dance-based interventions effects described as large (57, 58). Zumba dance, compared to treadmill walking and control groups, also improves cognitive functioning (59). According to the Synchronicity Hypothesis of Dance, humans dance to enhance both intra- and interbrain synchrony (60). Dance engages both interoceptive and exteroceptive processes, in addition to the haptic, visual, and auditory processing, which influence group synchrony, with haptic coupling having the most general effect on synchrony during group dancing (61). Exertive and synchronised movement modulates pain and may be an effective group bonding activity (20). For example, in a nine-month group exercise programme, participants reported that the social connections created within the group helped them reduce isolation, frustration, and depression (55). These results are in line with the literature on movement synchrony, as moving in sync with others makes us happier and more connected. For example, in a study in which participants were walking together, greater coordination resulted in greater feelings of agency, strength, and happiness (15). Such synchronisation also occurs implicitly, since humans tend to sync their movements with others (15, 62).

Interpersonal synchrony occurs in many contexts, and not everyone finds group dance or yoga attractive. Paying more attention to personal factors in pain management interventions by including activities that, for example, offer enjoyable and meaningful connections within one's social networks, could lead to improvements in physical and psychological well-being (63)*.* Thus, it may be useful to study INS in people with chronic pain also during other types of social learning, such as learning to play music together with others or playing collaborative games (64).

## 5 Discussion

To the best knowledge of the author, there have been no studies on INS in people with widespread chronic pain. Therefore, this paper aims to spark interest in the pain research community in the role of interpersonal synchrony in chronic pain treatment. Importantly, the field of INS research is still relatively young and thus, faces the challenges of defining INS and forming a uniform theoretical framework which would explain the effects described in the literature (9). Therefore, one may only speculate on the precise role of INS in the alleviation of fibromyalgia symptoms and the restoration of the social bodily self. There are various types of interpersonal synchrony, and thus, the mechanisms underlying its health benefits in each case may vary. Nonetheless, one may suspect that recurringly switching attention from internal, painful to external, social stimuli could play a role in reducing the attentional bias towards painful stimuli, typical for PwF (65). Additionally, the health benefits may come from improved interoception, as social contact improves bodily self-awareness (29, 37). At last, may INS-enhancing activities feel good and make people bond (20, 23), which may improve psychological symptoms in PwF, who often suffer from social alienation and loneliness (66).

Among multiple ways forward, two main research pathways are emerging, related to (1) describing the mechanisms of INS in people diagnosed with fibromyalgia (and chronic pain in general), and (2) its potential role of modulating symptoms.

The first pathway to validate the proposed hypothesis would be to investigate whether such synchrony occurs similarly to the general population, and if not, what are the differences. For example, do higher pain levels impede INS, as one becomes too overwhelmed with their own bodily signals to direct their attention toward others? What types of activity evoke INS in PwF?

The second pathway is related to the modulating effect of interpersonal synchrony. In what circumstances can being with others distract someone from their own pain and other symptoms? Could such INS-inducing interventions help modulate fibromyalgia symptoms and which symptoms should be targeted? What frequency and duration are needed to observe the alleged benefits? These are just a few of multiple questions that need to be answered to establish what role INS can play in fibromyalgia treatment and, more broadly, chronic pain management.

There are also several important challenges that need to be addressed. First, although significant progress has been made in the last decade (51), the underlying mechanisms of fibromyalgia itself are very complex and not fully understood, leading to a large heterogeneity of the population due to diagnostical difficulties (67). Other challenges are related to the character of symptoms, which hinder adherence to treatments, as the burden of commuting to regularly participate in treatment sessions is very high (68), which could potentially be reduced by designing interventions using virtual or extended reality (69, 70).

### 5.1 Conclusions

This article briefly introduced the concept of interpersonal neural synchrony and linked it for the first time to studies on fibromyalgia and its treatment. Various types of interventions that can involve interpersonal synchrony, such as group dance or exercise, have been shown to be effective in lasting symptoms reduction in people diagnosed with fibromyalgia. Future research is necessary to describe the INS mechanisms in fibromyalgia and reveal their potential health benefits, to verify whether they can eventually contribute to the reconnection on both intra- and interpersonal levels for people living with this severe condition.

## Data Availability

The original contributions presented in the study are included in the article/Supplementary Material, further inquiries can be directed to the corresponding author.

## Footnotes

^1^The European Alliance of Associations for Rheumatology, https://www.eular.org/
